# Episomal Induced Pluripotent Stem Cells Promote Functional Recovery of Transected Murine Peripheral Nerve

**DOI:** 10.1371/journal.pone.0164696

**Published:** 2016-10-13

**Authors:** Charles Yuen Yung Loh, Aline Yen Ling Wang, Huang-Kai Kao, Esteban Cardona, Sheng-Hao Chuang, Fu-Chan Wei

**Affiliations:** 1 Vascularized Composite Allotransplantation Center, Chang Gung Memorial Hospital, Taoyuan, Taiwan; 2 Department of Plastic Surgery, Chang Gung Memorial Hospital, Taoyuan, Taiwan; 3 Graduate Institute of Clinical Medical Sciences, Chang Gung University, Taoyuan, Taiwan; 4 Division of Surgery and Interventional Science, University College London, London, United Kingdom; 5 College of Medicine, Chang Gung University, Taoyuan, Taiwan; University of Texas at Austin Dell Medical School, UNITED STATES

## Abstract

Traumatic peripheral nerve neurotmesis occurs frequently and functional recovery is often slow and impaired. Induced pluripotent stem cells (iPSCs) have shown much promise in recent years due to its regenerative properties similar to that of embryonic stem cells. However, the potential of iPSCs in promoting the functional recovery of a transected peripheral nerve is largely unknown. This study is the first to investigate *in vivo* effects of episomal iPSCs (EiPSCs) on peripheral nerve regeneration in a murine sciatic nerve transection model. Episomal iPSCs refer to iPSCs that are generated via Oct3/4-Klf4-Sox2 plasmid reprogramming instead of the conventional viral insertion techniques. It represents a relatively safer form of iPSC production without permanent transgene integration which may raise questions regarding risks of genomic mutation. A minimal number of EiPSCs were added directly to the transected nerve. Functional recovery of the EiPSC group was significantly improved compared to the negative control group when assessed via serial five-toe spread measurement and gait analysis of ankle angles. EiPSC promotion of nerve regeneration was also evident on stereographic analysis of axon density, myelin thickness, and axonal cross-sectional surface area. Most importantly, the results observed in EiPSCs are similar to that of the embryonic stem cell group. A roughly ten-fold increase in neurotrophin-3 levels was seen in EiPSCs which could have contributed to peripheral nerve regeneration and recovery. No abnormal masses or adverse effects were noted with EiPSC administration after one year of follow-up. We have hence shown that functional recovery of the transected peripheral nerve can be improved with the use of EiPSC therapy, which holds promise for the future of nerve regeneration.

## Introduction

Trauma results in the majority of peripheral nerve injuries clinically. Direct lacerations and injuries often cause transection of peripheral nerves. Neurotmesis injuries represent the most severe form of peripheral nerve injury where total disruption of nerve fibers occur. Surgical coaptation of severed nerve endings is currently the mainstay form of treatment in order to facilitate axonal regeneration [1]. The function of transplanted limbs or faces in vascularized composite allotransplantation patients also depends on the speed of peripheral nerve recovery once coaptation of the recipient nerve is performed to the donor nerve [2]. However, recovery of completely transected peripheral nerves is often slow and can result in a delay of axonal regrowth to end target organ motor plates [3]. This causes muscle atrophy and functional impairment. Hence, strategies for improving the speed at which axons regenerate are crucial in restoring functional outcomes in patients with such traumatic injuries.

Induced pluripotent stem cells (iPSCs) have shown much promise in recent years due to its regenerative properties similar to that of embryonic stem cells (ESCs). The pluripotent nature of these cells allows them to differentiate into any somatic cell type in the body, allowing them to replace and rebuild for therapeutic purposes [4]. iPSCs can be reprogrammed from a patient’s own somatic cells, which makes them autologous in nature and reduces the risk of rejection [5]. They also carry fewer ethical considerations compared to embryonic stem cells which cause controversy [6].

iPSCs can be reprogrammed from somatic cells using several methods and these can be broadly classified into integrative and non-integrative methods [7]. Original reprogramming of somatic cells involves the in *trans* expression of pluripotency-related transcription factors namely Oct3/4, Klf4, Sox2 and c-Myc (OKSM). Integrative methods of reprogramming involve the use of viral delivery systems such as Moloney Murine Leukemia Virus-derived retroviruses, lentiviruses and adenoviruses which are used to carry OKSM pluripotent transgenes that incorporate permanently into the genome. These techniques carry the risk of viral transgene reactivation during differentiation of iPSC-derived cells which lead to the risk of genomic mutation [8]. Potent unwanted viral particles may carry oncogenes such as c-Myc. Furthermore, randomly distributed viral transgene insertions may result in the inactivation of host tumor suppressor genes or activation of oncogenes [9]. As such, iPSCs generated from these viral systems may limit their use.

Non-integrative methods, however, do not result in the integration of the OKSM pluripotent genes permanently into the genome. The deliberate exclusion of c-Myc in the reprogramming transgene further decreases the malignant transformation risk of the iPSCs produced. Transient episomal delivery systems using OKS plasmids provides a non-integrative method of iPSC production. The episomal vector carrying OKS genes provided by Shinya Yamanaka initiate the reprogramming process and are not integrated into the host’s genome [10, 11]. The episomal nature of these plasmids means that they are subsequently lost by plasmid dilution. iPSCs with improved safety profiles are produced as potential oncogenes are not introduced or perpetuated via genomic integration. As such, the iPSCs used in our study are termed episomal iPSCs (EiPSCs) to distinguish them from other methods of iPSC production.

There is a paucity of literature exploring the role of iPSCs on peripheral nerve regeneration. Furthermore, there are no studies that investigate the impact of EiPSCs on peripheral nerve regeneration. Our study is the first to demonstrate the effect of EiPSCs on the improvement in rate and degree of functional recovery of transected peripheral sciatic nerves in mice.

## Materials and Methods

### Mice

C57BL/6 (B6) female mice (6–8 weeks old) were purchased from the National Laboratory Animal Center, Taiwan. All murine procedures were carried out in full compliance with the recommendations in the Guide for the Care and Use of Laboratory Animals of the Chang Gung Memorial Hospital Animal research guidelines. Animal protocols were approved by the Committee on the Ethics of Animal Experiments of the Chang Gung Memorial Hospital (CGMH) in Taiwan and Institutional Animal Care and Use Committees (IACUC) of CGMH in Taiwan under permit numbers IACUC2014032502 and IACUC2016031109. The mice were housed in an environment enriched with chewing sticks and nesting material. The mice were monitored twice daily postoperatively and mice that had severe drop in body weight of 20–25%, displayed inability to seek food and water and inactivity were euthanized. No such adverse effects were noted in all of the animal experiments carried out in this study.

### Generation of EiPSCs

Mouse Embryonic Fibroblasts (MEFs) were transfected with pCX-OKS-2A plasmids using lipofectamine 3000 (Thermo Fisher Scientific, Waltham, MA). The plasmids were a gift from Shinya Yamanaka (Addgene plasmid #19771). The transfected MEFs were then cultured in iPSC medium and harvested as per the protocol stated (10). Embryonic stem cells of the C57BL/6 and 129 strain and MEF, OP9 stromal cells were obtained from ATCC.

### Real-time polymerase chain reaction (qPCR)

After culturing a similar number of EiPSCs, 129 ESCs and MEFs cells *in vitro*, RNA within each group of cells was extracted and converted to complementary DNA (cDNA). The expression of mRNA for Oct3/4, Sox2, Klf4 and Nanog in all stem cells was analyzed with TaqMan gene expression assays (Thermo Fisher Scientific, Waltham, MA). Respective levels of glial-derived neurotrophic factor (GDNF), neurotrophic growth factor (NGF), brain-derived neurotrophic factor (BDNF) and neurotrophic factor 3 (NT-3) in each group of cells were also quantified using qPCR. The expression in these experiments was normalized to that of RN18S.

### Flow cytometry analysis

Intracellular staining of pluripotent stem cells was performed by first fixing them in 70% ethanol for 16–18 hours at 4°C. Permeabilization of the stem cells was then carried out using 0.1% of Triton in PBS. Staining was then commenced using antibodies such as Sox-2, Nanog, Oct3/4 and isotype conjugated with Alexa Fluor^®^ 488 for flow cytometry that were purchased from BD Biosciences (Franklin Lakes, NJ).

### Immunocytochemistry

The stem cells were first cultured on coverslips. They were then fixed using a 4% paraformaldehyde in PBS and incubated for 20 minutes at room temperature. Primary antibodies such as Sox-2 (1:1000; abcam, Cambridge, MA), Oct-4A (1:400; Thermo Fisher Scientific, Waltham, MA), Nanog (1:50; Thermo Fisher Scientific, Waltham, MA) and β-actin (1:400; Sigma, St. Louis, MO) were added to the stem cells. A secondary antibody termed Rhodamine-labeled Goat anti-rabbit IgG was then added ontop. Nuclei were visualized with DAPI (4′6-diamindino-2-phenylindole dihydrochloride; 1:5000; Sigma, St. Louis, MO). Images were obtained using a Laser Scanning Confocal Microscopy (Leica TCS SP8X). On the other hand, alkaline phosphatase live staining reagent (Thermo Fisher Scientific, Waltham, MA) was added to the stem cells and incubated for 30 minutes at 37°C. The images were then obtained using a fluorescence microscope.

### Sciatic nerve transection model

Anesthesia was maintained with inhaled isoflurane. The posterior surface of the right hindlimb was shaved and prepped with 75% alcohol. The right sciatic nerve was then exposed via separating the gluteal musculature along a fascial plane. Division of the nerve was performed at a mid-thigh level, 1 cm proximal to the trifurcation of the nerve. Sharp division was conducted with microsurgical scissors and epineural repair was performed using 10–0 nylon suture. Four evenly spaced sutures were carefully placed in the epineural layer only for coaptation of both ends without any gaps.

### Application of EiPSCs, ESCs and buffer solution

A suspension of EiPSCs was added to a pocket created from the separation of gluteal muscles, immersing the transected sciatic nerve in the cells. This method of cell delivery is termed a topical application which reflects the method of administration. The muscles were then restored to their original positions and the skin was sutured closed. The same number of stem cells (5 x 10^5^) in each group were added to a similar PBS suspension for each of the experimental groups. The negative control group had PBS added alone without any cells.

### Group design

Four study groups were designed, namely, a group with EiPSCs added, another with C57BL/6 ESCs, a group with ESCs of the 129 strain and a negative control group with PBS added instead. Negative control and EiPSC groups had 15 mice each. B6 ESC and 129 ESC groups each had 6 mice. All groups had the right sciatic nerve transected at the same level and conditions between groups were kept similar for comparison.

### Functional Recovery Analyses

#### Five toe spread

Each mouse had their five toe spread distance measured prior to transection of the right sciatic nerve. Individual five toe spread distances were then measured at various time points during the course of the experiment. The recordings were repeated four times per mouse. The distance measured was expressed as a percentage of the distance pre-transection to indicate the degree of nerve regeneration represented by the five toe spread reflex and the degree of recovery of the intrinsic muscles of the foot innervated by the sciatic nerve [12, 13].

#### Video gait analysis

To evaluate the functional recovery of the sciatic nerve, an objective measurement of recovery was conducted via video gait analysis [13]. Video gait analysis is non-invasive, most accurately reflects and correlates with the quantitative measurement of isometric tetanic muscle force, which is an indication of sciatic nerve recovery itself [14]. Mice were observed walking along a walking track apparatus and filming of their movement was recorded using a 60Hz digital image camera. The recordings were repeated four times per mouse.

The ankle angle was measured between the leg and foot of the mouse. Four specific stages during the gait cycle were measured—1) initial contact when the right hindlimb touches the ground 2) midstance, where the contralateral foot crosses the experiment right hindlimb side 3) the toe off phase, where maximum plantar flexion of the foot on the experimental side is seen and 4) mid swing, where the experimental side foot crosses the contralateral foot. The resulting ankle angles were plotted against time and compared with each group. This not only represents the degree of sciatic nerve recovery, but also the rate and degree of muscle contracture.

#### Stereographic analysis of sciatic nerve regeneration

The right sciatic nerve was harvested from the transected area to the trifurcation of the nerve. Each group had a cross section analysis at a fixed distance (7 mm) distal from the point of transection of the sciatic nerve. A separate proximal cross section analysis was performed at the midpoint (3.5 mm) between the point of nerve coaptation and the point of distal cross section analysis. Toluidine blue staining and cross section axonal counts were performed to examine the level of axonal regeneration between each group [15]. Ultrathin sections (60 nm) were lifted onto formvar-coated grids, post-stained with lead citrate and uranyl acetate, and subsequently imaged using electron microscopy. A stereographic analysis of sciatic nerves from each group was conducted [16]. The density of the axons regenerated, as well as the level at which they regenerated to, were measured from the transected area and this was used to calculate the rate of nerve regeneration. A g-ratio for sciatic nerves in both groups at proximal and distal sections were performed. The g-ratio is estimated by dividing the axon diameter by the myelinated fiber diameter and it provides a method of evaluating nerve conduction velocity and fiber morphology during peripheral nerve regeneration.

#### Statistical analysis

Data was expressed as mean ± SEM and significance were evaluated by the two-tailed Student’s *t-*test. *P* < 0.05 was considered statistically significant. Differences between multiple groups in the recovery of five toe spread and gait were analyzed using a one-way analysis of variance (ANOVA). *P* < 0.05 was considered to be statistically significant. All calculations were conducted using SPSS 17.0 software.

## Results

### Somatic fibroblasts are successfully reprogrammed into EiPSCs

**Fig 1:**
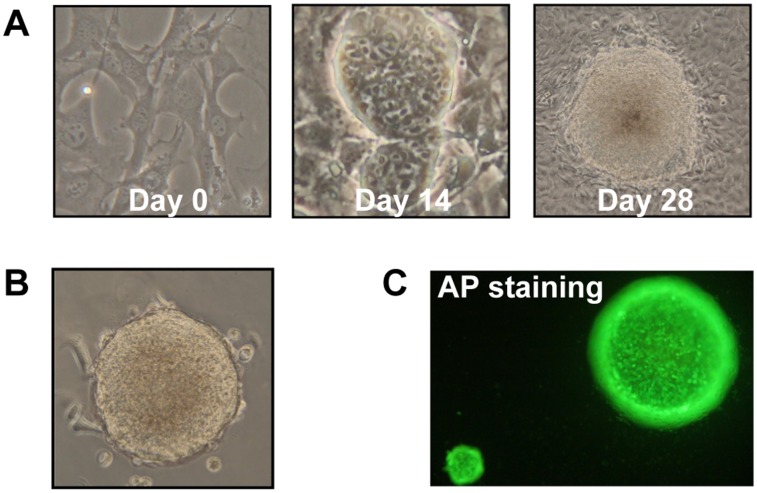
Reprogramming of EiPSCs. (A) Phase contrast images of the EiPSCs reprogrammed from MEFs at days 0, 14 and 28. (B) Microscopy images of embryoid bodies formed from EiPSCs. (C) EiPSC spheres stained for alkaline phosphatase (AP).

In order to explore the effects of EiPSCs on the regeneration of transected murine sciatic nerves, we set out to reprogram murine fibroblasts into EiPSCs. Reprogramming of C57BL/6 MEFs was first performed using Oct3/4-Klf4-Sox2 plasmids provided by Shinya Yamanaka [10]. We initially observed a distinct change in fibroblast cell morphology. The spindle-shaped fibroblasts gradually changed in shape to become more ovoid in shape. The ovoid cells were self-renewing in nature which then proceeded to form 3D spherical structures (Fig 1A).

These spherical structures were then selected and dissociated into single cells. The single cells then spontaneously self-renewed and formed embryoid bodies in an anti-differentiation suspension medium which were characteristic of pluripotent stem cells (Fig 1B).

The spheres stained for alkaline phosphatase (AP), a characteristic stain for pluripotent stem cells which confirmed their presence (Fig 1C). The cells within the spheres all indicated positive staining for AP.

These reprogrammed cells displayed promise in self-renewal capabilities, which suggest the generation of EiPSCs. Further characterization of these cells will determine the exact pluripotency of the cells.

### EiPSCs generated display characteristic pluripotent gene expression

**Fig 2:**
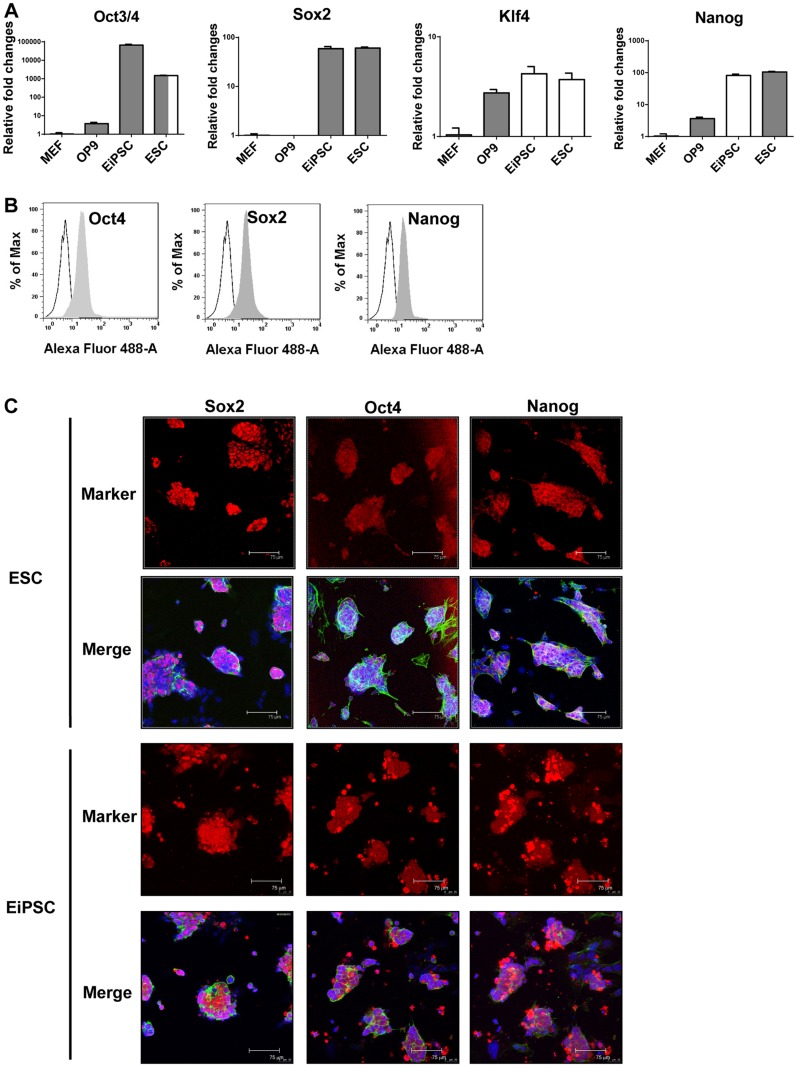
Characterization of EiSPCs. (A) The mRNA expression levels of pluripotent genes were quantified by real-time PCR in MEFs, OP9, EiPSCs and ESCs. (B) The pluripotent protein levels within EiPSCs were quantified using flow cytometry analysis. (C) Confocal microscopy images of immunocytochemistry staining for pluripotent protein expression in both EiPSCs and ESCs. Red fluorescence represents pluripotent protein expression whereas blue fluorescence represents DAPI staining for cell nuclei. Green fluorescence represents β-actin staining. Each scale bar represents 70 μm.

To further confirm the nature of the EiPSCs generated, the mRNA and protein expression levels of pluripotent genes were quantified in the reprogrammed cells. EiPSCs interestingly presented with similar levels of Oct3/4, Sox2, Klf4 and Nanog gene expression when compared to ESCs. In contrast, somatic cells such as MEFs and OP9s had minimal pluripotent gene expression (Fig 2A).

Intracellular staining for pluripotent proteins in EiPSCs was performed. Flow cytometry was used to accurately quantify levels of Oct4, Sox2 and Nanog. A complete shift in the peak for the pluripotent proteins compared to isotype control demonstrated greater than 90% high-level Oct4, Sox2 and Nanog expression within EiPSCs. This significantly proves the presence of pluripotent proteins within the generated EiPSCs (Fig 2B).

Immunocytochemistry staining for pluripotent proteins within the generated EiPSCs was conducted. Cells nuclei were stained with DAPI and pluripotent proteins were represented by red fluorescence to determine their locations. EiPSCs showed strong red fluorescence which is indicative of pluripotent gene expression at a similar level to ESCs (Fig 2C).

The results above demonstrated the successful reprogramming of somatic MEFs into EiPSCs with pluripotent gene expression similar to that of ESCs.

### EiPSCs facilitate faster recovery of the five toe spread reflex

**Fig 3:**
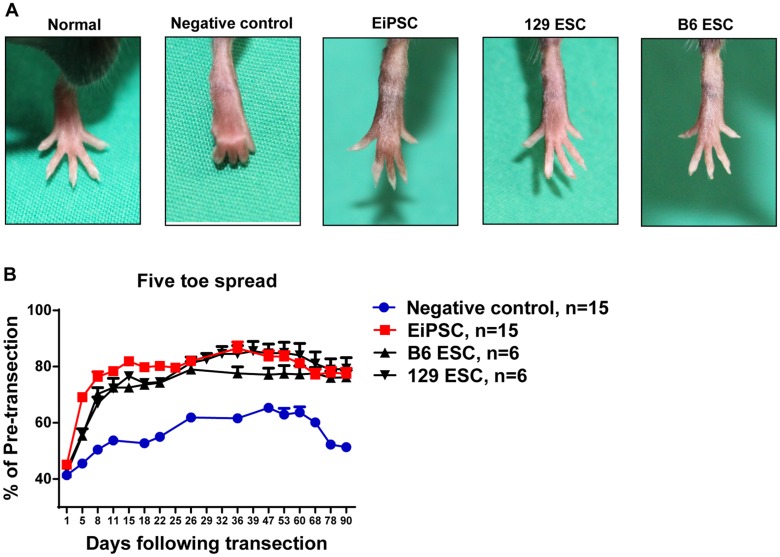
Functional recovery evaluated using five toe spread analysis. (A) Photographs of five toe spread at postoperative day 36 in five different groups of mice—normal (pre-transected), negative control (PBS only), EiPSC, 129 ESC and C57BL/6 (B6) group. (B) Cumulative graph of the four different groups expressed as a percentage of five toe spread recovery over pre-transection measurements postoperatively. The statistical data comparing EiPSC, 129 ESC and B6 ESC groups between negative control showed a mean±SEM which was significant (*P* value of < 0.0001, one-way ANOVA) at each time point.

To determine the effects of EiPSCs on the functional recovery of transected sciatic nerves, we utilize a well-known method of functional monitoring of sciatic nerve recovery in mice [12, 13]. Five toe spread measurements were recorded weekly in order to assess the rate and degree of sciatic nerve recovery. Faster rates of nerve recovery result in a stronger recovery of intrinsic muscles in the foot, thus allowing greater toe spread to happen. Almost full abduction of the toes was seen in all the three stem cell groups that were similar to non-transected toes. The negative control group however at any reference time period displayed a smaller degree of five toe spread. In some mice, clawing of the toes occurred and was associated with a fixed flexion contracture of the toe joints as seen in the negative control photograph (Fig 3A).

Faster re-innervation seen in the EiPSC group resulted in a larger toe spread compared to the negative control group. The EiPSC group also displayed an accelerated and enhanced five toe spread recovery. This recovery curve was similar to that of the C57BL/6 ESC and 129 ESC groups. A sustained recovery to about 80% of the pre transected five toe spread distance one month after transection was observed in the EiPSC, ESC and 129 ESC groups. The negative control group, however, saw an initial slower recovery in five toe spread and a maximum of 60% of the pre-transected distance before falling off. There was eventual clawing and permanent contracture formation of the toes when nerve recovery failed to occur completely. Probable motor endplate degeneration occurred on the intrinsic muscles as a resulting in atrophy. The resulting overall five toe spread measurement in the EiPSC group was larger than the negative control group, due to the greater recovery of sciatic nerve fibers within the same period of time before end motor plate atrophy. The results here demonstrated that EiPSCs show promise in improving the recovery of even the most distal of muscles (Fig 3B).

This is clinically relevant and also mimics clinical scenarios where muscle atrophy occurs and resulting contracture over joints form when intrinsic muscles are denervated. If nerve recovery is accelerated, motor end plates can be preserved and eventual functional outcomes can be maintained.

### EiPSCs promote the restoration of normal gait

Maintenance of muscle bulk correlates with nerve recovery and contributes to the functional outcome of sciatic nerve recovery. Utilizing video gait analysis, assessment and monitoring of sciatic nerve recovery in mice has been well documented as a measure of functional recovery, especially in the form of muscle bulk maintenance [13, 14].

There are four stages during the murine gait cycle, namely the initial contact where the foot touches the ground, the midstance where the contralateral foot crosses the experimental leg, the toe-off phase where there is maximum plantar flexion of the experiment side’s ankle joint and lastly the mid-swing phase where the experimental side foot crosses the contralateral foot. In particular, two stages during the murine gait cycle have been proven to correlate with isometric muscle force generated. During the toe-off phase, adequate strength has to be generated in order for the body weight of the mouse to be supported and provide lift off. By measuring the ankle angles, larger angles indicate better muscle recovery. With denervated muscle, the angle formed becomes smaller over time, with eventual contracture formation.

**Fig 4:**
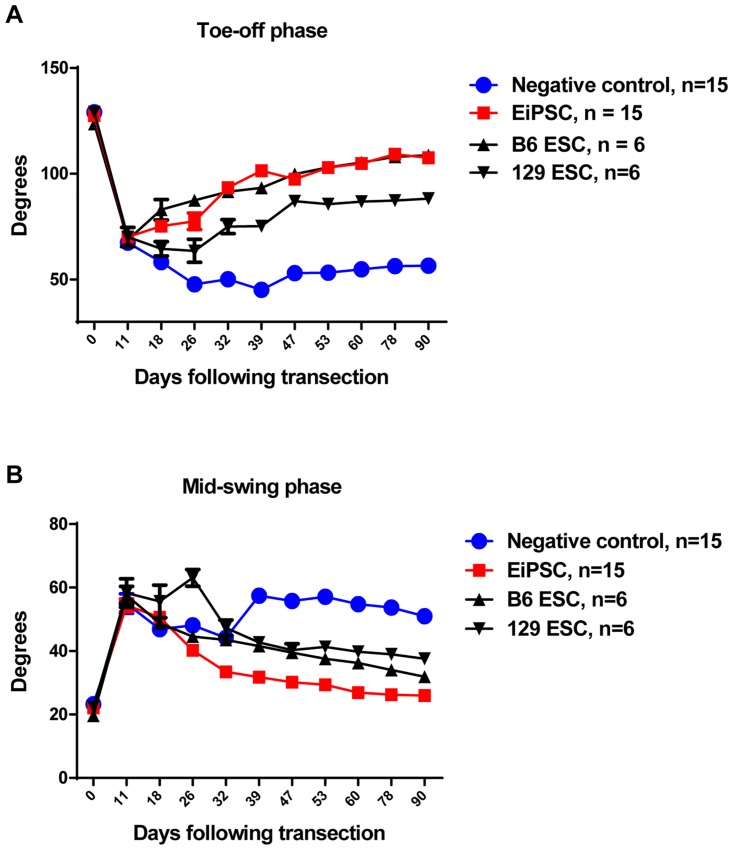
Functional recovery evaluated using video gait analysis. (A) Ankle angles during the toe-off phase post sciatic nerve transection in four groups. The statistical data comparing EiPSC, 129 ESCs and B6 ESCs groups between negative control showed a mean±SEM difference which was significant (*P* value of < 0.0001, one-way ANOVA). (B) Ankle angles during the mid-swing phase post sciatic nerve transection in four groups. The statistical data comparing EiPSC, 129 ESCs and B6 ESCs groups between negative control showed a mean±SEM difference which was significant (*P* = 0.0063, one-way ANOVA).

All groups showed a sharp decrease in toe off angles initially before gradually recovering. EiPSC, C57BL/6 ESC and 129 ESC groups all demonstrated a significant (*P* value of < 0.0001, one-way ANOVA) increase in angles at a quicker rate compared to negative control (Fig 4A).

The final stage of the gait cycle ends with the mid-swing phase. The ability to sustain a flexed ankle during gait is indicative of adequate muscle recovery. A normal mouse would adequate power to flex the ankle would have a smaller angle. When denervated, the ankle angle enlarges as there is a lack of muscle power to lift the leg. The EiPSC group demonstrated initially widening of ankle angles during this phase but as recovery took place, showed the largest decrease in ankle angles. This pattern was similar to the C57BL/6 ESC and 129 ESC groups. The negative control group, however, showed temporary improvement in ankle angles but failed to sustain this and were enlarged at the end (*P* value of = 0.0063, one-way ANOVA) (Fig 4B).

The results here indicate that faster sciatic nerve recovery allows for maintenance of end target muscle function. When nerve recovery is delayed, muscle atrophy will impair functional outcomes. From our study, EiPSCs have the potential to improve nerve recovery rates and ameliorate the loss of denervated muscle fibers.

### EiPSCs result in enhanced axonal regeneration

**Fig 5:**
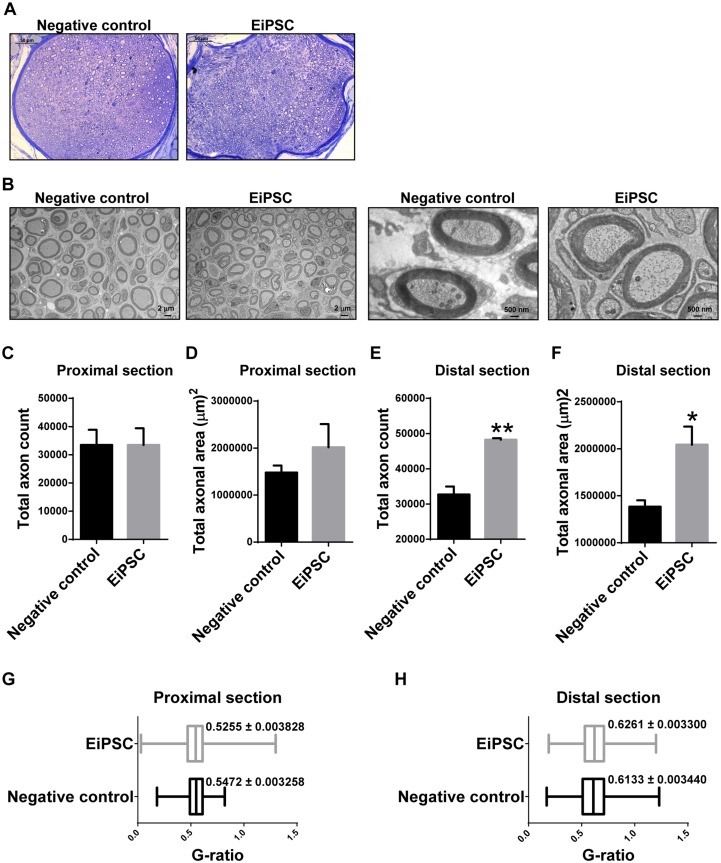
Stereographic analysis of sciatic nerve regeneration. (A) Toluidine blue staining of fascicles in the distal section of regenerated sciatic nerves. Each scale bar represents 50 μm. (B) Electron microscopy of regenerated axons in both negative control and EiPSC groups at the distal sections. The scale bars for two images on the left are 2 μm each and 500 nm for two images on the right. (C, E) Total axon count in the proximal (C) and distal (E) section of regenerated sciatic nerves. (D, F) Total axonal area in the proximal (D) and distal (F) section of regenerated sciatic nerves. (G-H) G-ratio comparing the proximal (G) and distal (H) section of regenerated sciatic nerves in both EiPSC and negative control groups.

To further obtain direct evidence and statistical quantification of the degree of sciatic nerve regeneration, mice in each group were sacrificed and stereographical analysis of the sciatic nerves was performed at a fixed distance away from the point of transection. Sciatic nerves had a greater number of axons in the EiPSC group compared to the negative control group (Fig 5A). A similar observation was seen on electron microscopy (Fig 5B).

Proximal section analysis of the regenerated sciatic nerve revealed a similar total axon count and total axonal area in both EiPSC and negative control groups (Fig 5C and 5D). With a short initial distance to recover, both groups demonstrated similar axonal regeneration.

However, with accelerated axonal regeneration and subsequent functional recovery of peripheral nerves, a greater number of axons would be expected to grow within the same time period in a nerve fiber. In the distal cross section analysis, a significantly increased (*P* = 0.0025, unpaired t-test) total number of axons in the EiPSC group was seen when compared to negative control in the entire sciatic nerve (Fig 5E).

The distal section total axonal surface area was correspondingly significantly higher (*P* = 0.0322, unpaired t-test) in the EiPSC group compared to the negative control group. With a greater number of axons and total surface area, increased conduction of impulses to the corresponding muscles occurs, thus eliciting a better response and resulting in better functional outcomes (Fig 5F).

The g-ratio evaluates the amount of myelination in relation to axon diameter which in turn, can be used to compare the degree of nerve regeneration. A lower g-ratio is indicative of a higher myelin to axon diameter ratio and vice versa. A lower g-ratio was seen in both EiPSC and negative control nerves proximally compared to distal sections which reflected a better nerve regeneration proximally than distally. The g-ratio in the proximal section of the EiPSC group was 0.02 lower than negative control (Fig 5G and 5H).

The axonal regeneration seen is related to our results seen in Figs 3 and 4 where five toe spread intrinsic muscle function is maintained. Recovery of muscle bulk during gait was also evident in the EiPSC group which suggests that EiPSCs maintain muscle bulk and function by promoting the increased recovery of the total number of axons distally within the transected sciatic nerve.

### EiPSCs may improve peripheral nerve regeneration through elevated levels of neurotrophin-3

**Fig 6:**
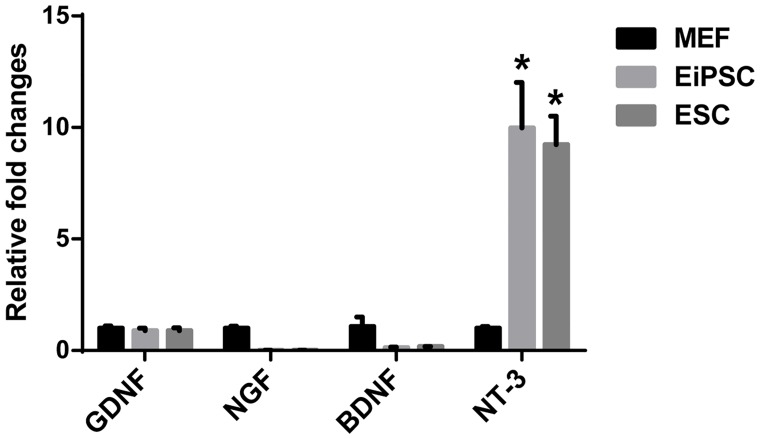
Quantification of stem cell derived neurotrophic factors. Chart comparing mRNA levels of glial-derived neurotrophic factor (GDNF), neurotrophic growth factor (NGF), brain-derived neurotrophic factor (BDNF) and neurotrophic factor 3 (NT-3) produced by MEF, EiPSCs and ESCs quantified using qPCR.

In order to shed light on possible mechanisms behind the effects of EiPSCs on promoting peripheral nerve regeneration, we examined the possible differences in neurotrophic factor levels of stem cells compared to regular MEFs. Neurotrophic factors, such as GDNF, NGF, BDNF and NT-3, act through possible paracrine pathways in influencing peripheral nerve regeneration and hence could account for the immediate differences seen between each group. When we compared EiPSCs and ESCs to MEF cells, the stem cells inherently display a significantly elevated (*P* = 0.0337, one-way ANOVA) level of NT-3 mRNA (Fig 6). NT-3 is encoded by the gene NT-3 and as a neurotrophic factor, is not only responsible for the survival of dorsal root ganglion (DRG) neurons but also is a chemoattractant for DRG axons [17, 18]. It also promotes the growth and differentiation of new neurons and synapses [19, 20]. The almost 10 fold increase in NT-3 mRNA expression in both EiPSCs and ESCs compared to MEFs may be partially responsible for their effects seen in our study. This suggests that EiPSCs accelerate peripheral nerve regeneration partially through paracrine effects via neurotrophic factors such as NT-3 in particular.

### EiPSCs appear to have no adverse effects in mice

**Fig 7:**
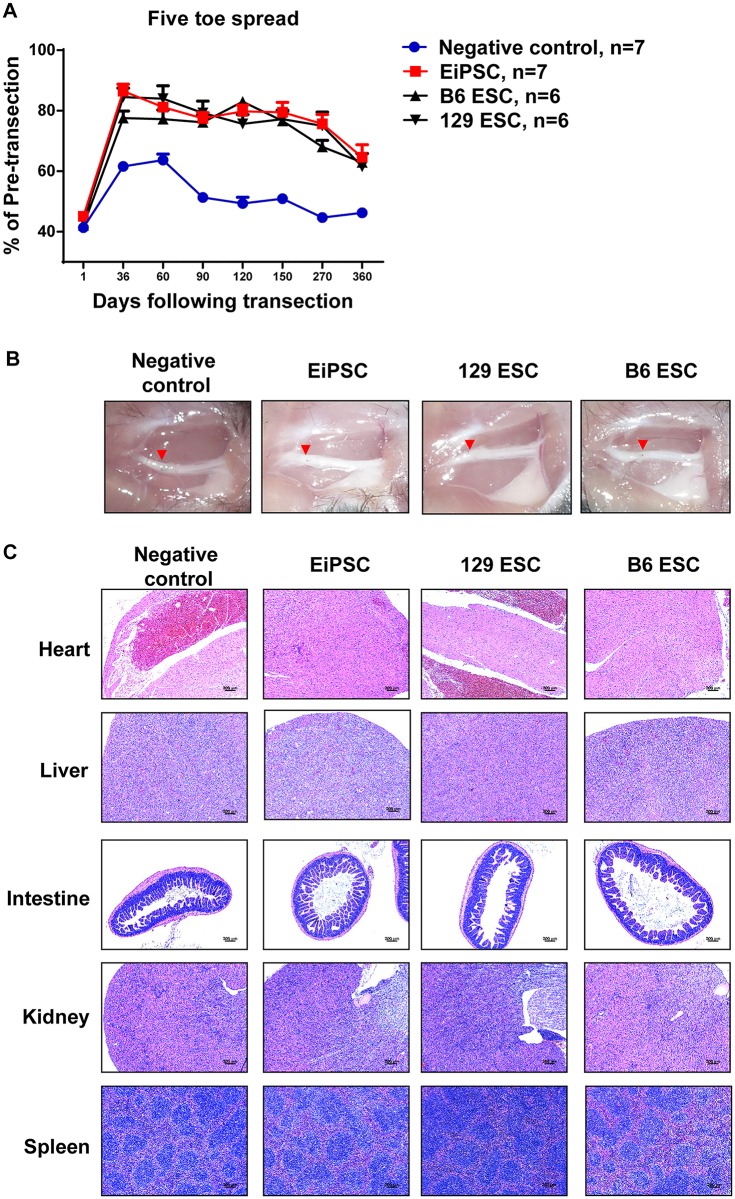
The safety profile of EiPSCs and functional recovery after one year of long-term follow-up. (A) Recovery of five toe spread of mice in each group over the course of one year (*P* = 0.0025, one-way ANOVA). (B) Macroscopic examination of the surrounding tissues including the site of nerve coaptation (red arrow) where stem cells or PBS were applied. (C) H&E pathological evaluation of various organs from mice in each group. Note the similar normal tissue architecture in (B) and (C) for each stem cell group compared to negative control.

Five toe spread measurements were performed for up to a year in each of the experimental groups. Even though all the stem cell groups displayed a similar trend with eventual reduction of five toe spread distance after one year, the stem cell groups maintained a greater degree of five toe spread compared to negative control after one year, which is indicative of better intrinsic function recovery (Fig 7A).

Mice in each group were followed up for one year before they were sacrificed for both macroscopic and microscopic examination at the site of stem cell application as well as its systemic effects. Normal tissue architecture was observed macroscopically at the site of nerve coaptation and its surrounding tissue in each group (Fig 7B). Normal tissue architecture was also seen on H&E examination of various organs from mice in each group (Fig 7C) and a summative table of the findings are presented in Table 1. No teratoma formation or abnormal masses were found and the mice were noted to be healthy and active before they were sacrificed. Long-term follow-up with macroscopic and microscopic examination of the immediate tissue vicinity where EiPSCs were applied, as well as the critical organs of the mice revealed no gross abnormality. Coupled with the normal behavior observed in these mice, EiPSC administration has not resulted in any noticeable adverse effects after one year of follow-up.

**Table 1:** One year H&E pathological evaluation in organs of mice from the sciatic nerve model with various types of pluripotent stem cells.

	Negative control (N = 8)	EiPSC (N = 8)	129 ESC (N = 6)	B6 ESC (N = 6)
Heart	-	-	-	-
Liver	-	-	-	-
Intestine	-	-	-	-
Kidney	-	-	-	-
Spleen	-	-	-	-

“ - ” represents negative pathological findings on microscopic examination of various organs.

## Discussion

The topical application of EiPSCs after repair of transected murine sciatic nerves results in a significantly accelerated rate and degree of nerve recovery compared to the negative control group. An improved functional outcome was observed not only in terms of nerve recovery but also functional muscle outcomes. The end result is the culmination of the regenerative properties of EiPSCs in response to trauma from the initial sciatic nerve transection. This was evident from ESCs as well in our study which highlights the similar pluripotent properties of both EiPSCs and ESCs.

The results seen here are a novel preliminary look at the effect of EiPSCs on nerve recovery of coapted axotomized peripheral nerves. This mimics the clinical scenario where transection of nerves occur and surgical repair is performed. Peripheral nerve recovery is then slow and often unsatisfactory, leading to poor functional outcomes. As compared to other studies in the literature that focus on nerve structure regeneration and tissue engineering [21–24], the use of EiPSCs in this study was to significantly facilitate the recovery of peripheral nerves and axonal regeneration. Neurotrophic factors are essential in peripheral axonal growth and regeneration. BDNF [25] and GDNF are required in order to sustain axonal regeneration. An upregulation of these factors has been shown to accelerate axonal growth towards distal nerve ends [26]. In particular, the production of nerve growth factor, BDNF, GDNF, NT-3 and ciliary neurotrophic factors have been demonstrated by transplanted stem cells such as adipose-derived stem cells [27–31]. Hence, a possible partial explanation for the results seen is that EiPSCs result in an increase in neurotrophic factors locally at the site of application. NT-3, in particular, may be responsible for the promotion of murine sciatic nerve recovery.

Schwann cells (SC) are the principal glial cells of the peripheral nervous system and facilitate axonal regeneration once transection occurs [32, 33]. When neural injury occurs, SCs bring about peripheral nerve regeneration through clearing distal nerve debris and recruitment of circulating macrophages to the zone of injury [34]. A delayed recovery of axotomized peripheral nerves results in motor endplate denervation and central body apoptosis [35]. A loss of Schwann cells happens as a result [36].

The preservation of muscle function and neuromuscular junctions also contribute to enhanced motor functional recovery. Studies have shown that ESC-derived motor neurons contribute to the preservation of muscle and neuromuscular junctions after peripheral nerve injury [22, 37]. Evidence shows that EiPSCs possess the capability to differentiate into motor neurons *in vitro* [38–40].

A preparation of an allogeneic stem cell bank will provide a readily available source of stem cells for therapeutic uses. This will reduce costs and production time compared to autologous sources. However, there are concerns regarding the alloreactivity of these stem cells when placed in a host, resulting in rejection and ultimate lack of stem cell function. In our study, one of the groups was the use of allogeneic ESCs from strain 129 which interestingly produced similar results when compared to autologous ESCs. Allogeneic ESCs may play a role in improving functional recovery when compared to autologous EiPSCs in murine sciatic nerve regeneration.

Further modifications to the reprogramming process of EiPSCs can be performed in order to improve their safety profile. Episomal reprogramming plasmids carrying a reporter gene can be used to reprogram somatic cells. Once reprogramming has taken place, purifying of EiPSCs without the inclusion of plasmids with reporter genes can be performed using a cell sorter, thus effectively filtering out EiPSCs carrying reprogramming plasmids. This would further decrease the malignant transformation risks of EiPSCs due to the complete removal of pluripotent transgenes which in turn, reduce the possibility of transgene overexpression.

The topical application of EiPSCs in this study is a novel experimental design which reflects its potential to improve and enhance peripheral nerve regeneration. The potential for allogeneic EiPSCs to exert similar effects to autologous ones also opens up the possibilities for widespread use of EiPSCs as cell therapy adjuncts.
